# Risk factors for peripheral arterial disease in elderly patients with Type-2 diabetes mellitus: A clinical study

**DOI:** 10.12669/pjms.36.6.2906

**Published:** 2020

**Authors:** Zhang Shou, Yongcai Zhao, Yan Zhang, Shaoqing Li

**Affiliations:** 1 Zhang Shou, Department of Endocrinology, Cangzhou Central Hospital, Cangzhou, Hebei Province, 061001, China; 2 Yongcai Zhao, Department of Endocrinology, Cangzhou Central Hospital, Cangzhou, Hebei Province, 061001, China; 3 Yan Zhang, Department of Endocrinology, Cangzhou Central Hospital, Cangzhou, Hebei Province, 061001, China; 4 Shaoqing Li, Department of Endocrinology, Cangzhou Central Hospital, Cangzhou, Hebei Province, 061001, China

**Keywords:** Peripheral arterial disease (PAD), Type-2 diabetes mellitus (T2DM), Risk factors, Elderly population

## Abstract

**Objectives:**

To determine risk factors for peripheral arterial disease (PAD) in elderly patients with Type-2 diabetes mellitus.

**Methods:**

The elderly patients with Type-2 diabetes treated in the Central Hospital of Cangzhou were enrolled and divided into PAD group and non-PAD group between October 2016 and November 2019, The data of the patients including age, gender, body mass index, blood pressure, hemoglobin A1c, high-density lipoprotein-cholesterol, low-density lipoprotein-cholesterol, total cholesterol, triglyceride, white cell count, lymphocyte count, high-sensitivity C-reactive protein, uric acid as well as living habits and complications of Type-2 diabetes mellitus were recorded to determine the risk factors for PAD.

**Results:**

One thousand four hundred seventy six (1476) patients were enrolled, in which 465 patients were included in group of PAD, and 1011 in non-PAD group. The univariate analysis revealed that the two groups significantly differed in age (p=0.003), course of T2DM (p=0.001), hypertension (p=0.006), smoking habits (p<0.001), hyperuricemia (p<0.01), high-sensitivity C-reactive protein (p<0.01), white cell count (p<0.001), lymphocyte count (p<0.001) and diabetic neuropathy (p<0.001). In the multivariate analysis, age (OR: 1.56, 95% CI: 1.21-1.89), smoking habit (OR: 1.37, 95% CI: 1.19-1.68), hypertension (OR: 1.44, 95% CI: 1.15-1.98), diabetic neuropathy (OR: 3.55, 95% CI: 2.14-4.29), high-sensitivity C-reactive protein (OR: 1.74, 95% CI: 1.39-2.61) and hyperuricemia (OR: 2.71, 95% CI: 1.66-3.87) were significant risk factors for PAD.

**Conclusions:**

Age, smoking habit, hypertension, diabetic neuropathy, high-sensitivity C-reactive protein and hyperuricemia were independent risk factors for peripheral arterial disease in elderly patients with Type-2 diabetes mellitus.

## INTRODUCTION

Type-2 Diabetes Mellitus (T2DM), a complex chronic metabolic disorder, is steadily increasing in the world, especially in elderly population.^1^ Through time, T2DM may lead to many complications. Peripheral arterial disease (PAD), defined as lower extremity arterial atherosclerosis precipitated by the obstruction of a peripheral arterial vessel,^2^ is a primary cause of morbidity and mortality in elderly population,^3^ also one of the important complications of T2DM. Some authors found its prevalence in T2DM was between 10% and 42%, significantly higher than those without.^4^ Accompanied with PAD, the risk of cardiovascular mortality, functional limitation, leg revascularization, and amputation is significantly increased, leading to worse outcomes in patients with T2DM.^5^

In addition, with the coming of aging society, more elderly may suffer from T2DM and PAD, which may affect the quality of life and impose a heavy burden on society adversely. Consequently, it is necessary to detect the risk factors for PAD in elderly patients with T2DM. In a study of 150 T2DM patients and 150 healthy controls, Soyoye and colleagues found PAD was associated with increasing age, male gender, obesity, and high C-reactive protein levels in people with T2DM.^6^ In another study of 280 patients with T2DM, Akalu found increasing age, high HbA1c, and being cigarette smokers increase the likelihood of developing PAD.^1^ However, the results of these studies are inconsistent, no definite conclusions are available. In addition, these studies recruited patients with different ages instead of elderly patients. Subsequently, the risk factors for PAD in elderly patients with T2DM are still unclear.

Therefore, in this study we reviewed the elderly patients with T2DM treated in our hospital between October 2016 and November 2019, and our objective was to determine the prevalence and risk factors of PAD in elderly patients with T2DM, to help physicians understand PAD and T2DM correctly.

## METHODS

A total of 1658 elderly patients (≥ 60 years old) with T2DM treated in the Central Hospital of Cangzhou were included between October 2016 and November 2019. The inclusion criteria were: 1) patients were diagnosed with T2DM; 2) patients aged ≥60 years, and 3) patients agreed to participate the study and signed informed consents. T2DM was diagnosed among patients who satisfy the World Health Organization criteria for diabetes and were treated initially using oral hypoglycaemic agents and/or insulin.^6^ To facilitate the study, the patients with severe heart failure or renal failure^7^ as well as those with febrile illness, casts, ulcers, and other conditions that may affect the levels of high-sensitivity C-reactive protein (hs-CRP) or interfere with examination of posterior tibial and dorsalis pedis arteries were excluded.^6^ This study was approved by the ethics committee of our hospital.(April 12, 2020)

PAD is defined as an ABI <0.9 in at least one leg, and the ABI was recorded as the ratio of the ankle systolic blood pressure to the brachial systolic blood pressure, which were measured using Doppler blood stream probe^8^. The normal value of ABI is between 0.90 and 1.40. Those patients with an ABI >1.40 in any leg were excluded, because they may have arterial stiffness, and were difficult to assess PAD accurately.^8^,^9^ After the measurement of PAD, the participants were divided into PAD group and non-PAD group.

At the beginning of the study, physical examination was carried out for each participant, and the basic clinical data including age, gender, body mass index, and blood pressure were recorded in details, in which body weight and height were measured to the nearest 0.1cm and 0.1 kg, and body mass index was calculated as weight divided by height squared (kg/m^2^), blood pressure was recorded using a mercury sphygmomanometer, and the definition of hypertension is systolic blood pressure (SBP)≥140mmHg or diastolic blood pressure (DBP) ≥ 90mmHg. In addition, a blood sample specimen from antecubital vein was obtained in each participant after 10-12 hour overnight fasting for the analysis of hemoglobin A1c (HbA1c), high-density lipoprotein-cholesterol (HDL-C), low-density lipoprotein-cholesterol (LDL-C), total cholesterol (TC), triglyceride (TG), hs-CRP and uric acid, which were measured using an automatic analyzer.^1^ In addition, the information including living habits such as smoking history and alcohol use as well as complications of T2DM were also recorded.

The data was analyzed using SPSS 23.0 (SPSS Inc., Chicago, IL, United States). The analysis of categorical variables was carried out using chi-squared test, measurement data was compared using analysis of variance, and univariate analysis were performed to detect the associations between variables and PAD, and the multivariate logistic regression analysis was employed to determine the independent risk factors for PAD. A p value < 0.05 was regarded as significance.

## RESULTS

In this study, 1476 patients were enrolled and 182 were excluded. Among the included patients, 465 patients were diagnosed with PAD and included in group of PAD, and the remaining 1011 patients were included in non-PAD group. The mean age in PAD group was 72.5 years, ranging from 60 to 89 years, and in non-PAD group was 66.3 years, ranging from 60 to 85 years. Two hundred thirteen (45.8 %) patients were male in PAD group and 425 patients (42%) were male in non-PAD group. In the current study, the prevalence of PAD was 31.5%. In PAD group, 165 patients (35.5%) were symptomatic, of which 98 patients had typical intermittent claudication, while 300 patients (64.5%) were asymptomatic.

**Fig.1:**
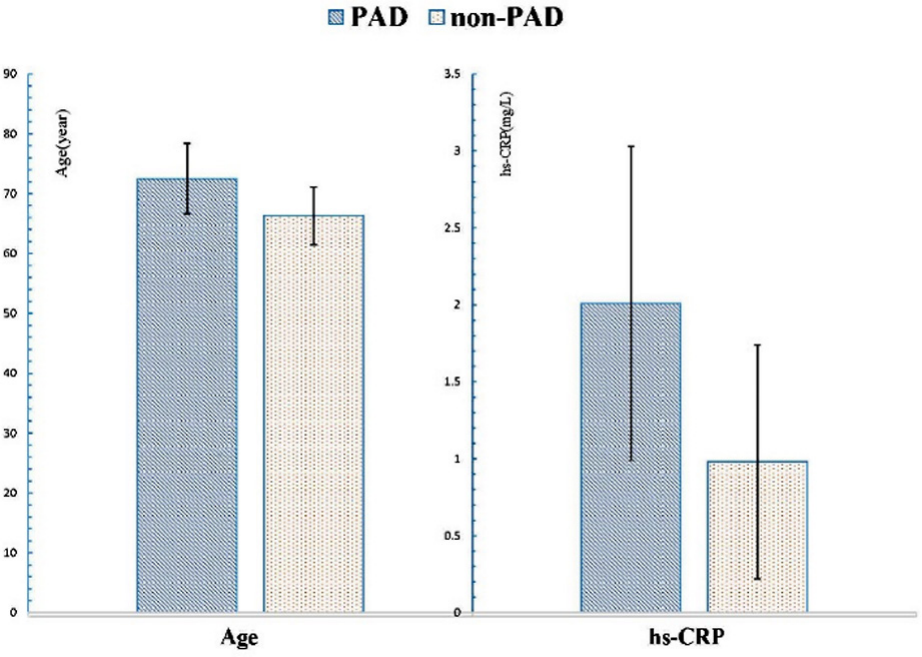
The comparison of age and hs-CRP between the two groups.

**Fig.2:**
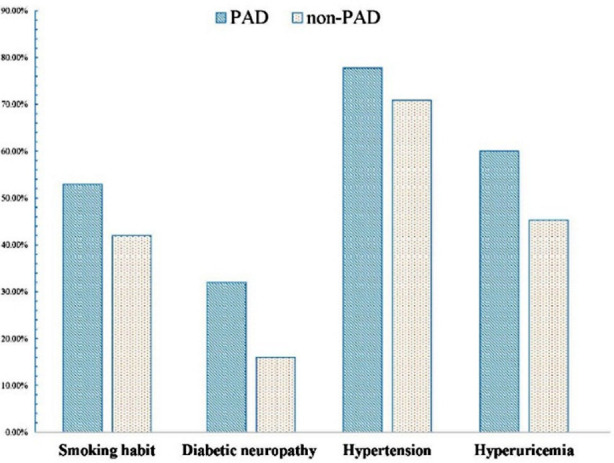
The comparison of smoking habit, diabetic neuropathy, hypertension and hyperuricemia between the two groups.

The clinical characteristics of patients with or without PAD show in Table I. The results of univariate analysis revealed the two groups significantly differed in age (p=0.003), course of T2DM (p=0.001), hypertension (p=0.006), smoking habits (p<0.001), alcohol use (p<0.001), hyperuricemia (p<0.01), hs-CRP (p<0.01), white cell count (p<0.001), lymphocyte count (p<0.001) and diabetic neuropathy (p<0.001), but did not in gender (p=0.19), BMI (p=0.29), HbA1c (p=0.069), hyperlipidemia (p=0.28), and diabetic retinopathy (p=0.67)(Fig. 1, 2). Patients in PAD group had older age, longer durations of T2DM, a higher prevalence of hypertension, diabetic neuropathy and hyperuricemia, a higher proportion of smoking habit and alcohol use, a higher value of hs-CRP, white cell count and lymphocyte count, compared with those in non-PAD group.

**Table-I:** Clinical characteristics of patients in the two groups

Variables	PAD group n=465)	Non-PAD group (n=1011)	p value
Age (Years)	72.5±5.9	66.3±4.8	0.003
Gender (Male/Female)	213/252	425/586	0.19
BMI(kg/m^2^)	25.7±3.4	24.6±3.5	0.29
Hypertension (n, %)	362 (77.8%)	717 (70.9%)	0.006
T2DM duration (Years)	15.6±5.7	11.5±4.6	0.001
HbA1c (%)	7.9±1.1	7.7±0.8	0.069
Hyperlipidemia (n, %)	284 (61.1%)	586 (57.9%)	0.28
Smoking habit (n, %)	246 (52.9%)	425 (42%)	<0.001
Alcohol use (n, %)	146 (32.0%)	253(25.0%)	<0.001
Diabetic retinopathy (n, %)	128 (27.5%)	266 (26.3%)	0.67
Diabetic neuropathy (n, %)	149 (32%)	162 (16%)	<0.001
Hyperuricemia (n, %)	279 (60%)	458 (45.3%)	<0.001
hs-CRP (mg/L)	2.01±1.02	0.98±0.76	<0.001
Total white cell count (×10^3^)	6.53±1.28	5.15±1.09	<0.001
Lymphocyte count	3.63±1.42	2.45±0.91	<0.001

Based on the outcome of the univariate analysis, multivariate analysis was carried out, and the OR values of the independent risk factors of PAD were calculated. The statistically significant risk factors for PAD in elderly patients in T2DM were age (OR: 1.56, 95% CI: 1.21-1.89), smoking habit (OR: 1.37, 95% CI: 1.19-1.68), hypertension (OR: 1.44, 95% CI: 1.15-1.98), diabetic neuropathy (OR: 3.55, 95% CI: 2.14-4.29), hs-CRP ( OR: 1.74, 95% CI: 1.39-2.61) and hyperuricemia (OR: 2.71, 95% CI: 1.66-3.87) (Table-II). In addition, in this study, significantly positive correlations were found among the values of blood pressure, uric acid, and hs-CRP in PAD group (p<0.05).

**Table-II:** The independent risk factors for peripheral arterial disease.

Factors	OR (95% CI)	p values
Age	1.56 (1.21-1.89)	0.006
Smoking habit	1.37 (1.19-1.68)	0.003
Diabetic neuropathy	3.55 (2.14-4.29)	0.005
Hypertension	1.44 (1.15-1.98)	0.011
Hyperuricemia	2.71(1.66-3.87)	0.001
hs-CRP	1.74 (1.39-2.61)	0.015

## DISCUSSION

We have tried to evaluate the risk factors of PAD in elderly patients with T2DM. To the best of our knowledge, few studies have been conducted on the issue. As to the screening of PAD, Ankle-brachial pressure index (ABPI) is a simple though validated screening parameter for identifying asymptomatic individuals with PAD. Some studies demonstrated that ABI of less than 0.9 is 95% sensitive and close to 100% specific in the diagnosis of PAD 3, so the cases in which the ankle-brachial systolic pressure index measurement for clinical diagnosis is below 0.9 are usually defined as PAD.^10^ ABI has a high sensitivity and specificity, combined with low costs, which make it work as an excellent screening test of choice all over the world. Subsequently, in this study we used ABI to diagnose PAD.

In the current study, the prevalence of PAD was 31.5%. Compared with previously published studies^1^,^6^, the rate of PAD is higher. In this study, only elderly patients were included, with age increasing the level of atherosclerosis increased significantly. Moreover, T2DM co-existed with other risk factors for cardiovascular disease such as hypertension and hyperuricemia, which adversely aggravate the development of PAD, leading to a higher prevalence of PAD in the current study. However, in a study of 200 patients with T2DM, Agboghoroma reported the prevalence of PAD was 38.5%, although in his study the age of patients ranged from 31 to 90 years.^11^ Compared with Agboghoroma’s study, the prevalence of PAD in our study was relatively low. We believe the difference may come from some factors such as sample size, inclusion criterion and race.

To fully understand the factors influencing the development of PAD, we tried to include more parameters, and found the independent risk factors for PAD in elderly patients with T2DM were age, smoking habit, hypertension, diabetic neuropathy, hs-CRP and hyperuricemia. Some of the parameters like age, smoking habit, hs-CRP and hypertension were consistent with the studies of Akalu^1^ and Soyoye.^6^ However, differently, we found obesity and gender were not the independent risk factors. In this study, only elderly patients were included, they usually have larger BMI values, which may lead to such a different outcome in the current study. In terms of gender, some other studies also found it had no significant relationship with PAD.^8^,^12^ Subsequently, among the studies available, the influence of gender on PAD seems still controversial. As most of the studies were cross-sectional studies, no longitudinal and randomized controlled studies were conducted. In addition, the influence of gender may be intervened by culture, life style, country and areas, race and many other factors. Hence, a rigorously designed randomized controlled trial with long-term follow-up may be helpful on the issue.

Moreover, hyperuricemia was also an independent risk factor. Some studies found that in patients with hyperuricemia, the accumulation of uric acid and activation of xanthine oxidoreductase may induce inflammation and increase oxidative stress, accelerates atherosclerotic progression, and promote the progression of PAD.^13^ In a study of 508 patients with T2DM, Tseng found uric acid levels were higher in patients with PAD than in those without, suggesting elevated uric acid level is a significant and independent risk factor for PAD in patients with T2DM.^9^ In Tseng’s study, some patients were not elderly, while they obtained the same conclusion. With the age increasing, the level of uric acid may increase. Subsequently, in the current study, it was reasonable that the level of uric acid is closely associated with the development of PAD in elderly patients with T2DM. Based on these two studies, we can conclude that hyperuricemia has similar influence on PAD in both elderly and younger patients with T2DM.

Diabetic neuropathy was also found to be an independent risk factor. Diabetic neuropathy is the most common microvascular complications of diabetes mellitus,^14^ and its development indicates T2DM begin to influence capillaries adversely. Consequently, it is reasonable that diabetic neuropathy is closely associated with the progression of PAD. In addition, positive correlations were found among the values of blood pressure, uric acid, and hs-CRP in PAD group. In a study of 67 hypertensive and 30 healthy subjects, Mutluay and colleagues found hyperuricemia is an independent predictor for early atherosclerosis in hypertensive subjects,^15^ and inflammation play an important role during the process of atherosclerosis, in which hs-CRP is an important marker. Our study further confirmed these viewpoints.

In brief, in this cross-sectional study with a large sample size, we found that age, smoking habit, hypertension, diabetic neuropathy, hs-CRP and hyperuricemia were independent risk factors for PAD in elderly patients with T2DM.

### Limitations of the study

In PAD group some patients were symptomatic and some were asymptomatic. If a comparison was performed between these patients, aditional information may be obtained to facilitate understanding of related risk factors, while the comparative study was not performed. In addition, this study further proved some viewpoints of previously published literature, but some controversial viewpoints are still available. Hence, more studies should be performed in the future.

### Authors Contribution:

**ZS, YCZ:** Designed the project and did statistical analysis,

**ZS, YCZ, YZ** **& SQL:** Did data collection and manuscript writing,

**ZS & YCZ:** Did review, final approval of manuscript and are responsible for integrity of research.
